# The Adaptive Neuroplasticity Hypothesis of Behavioral Maintenance

**DOI:** 10.1155/2012/516364

**Published:** 2012-10-16

**Authors:** Janey C. Peterson

**Affiliations:** Weill Cornell Medical College, Center for Integrative Medicine and the Division of Clinical Epidemiology and Evaluative Sciences Research, New York, NY 10065, USA

## Abstract

Physical activity is a seemingly simple and clinically potent method to decrease morbidity and mortality in people with coronary heart disease (CHD). Nonetheless, long-term maintenance of physical activity remains a frustratingly elusive goal for patients and practitioners alike. In this paper, we posit that among older adults with CHD, recidivism after the initiation of physical activity reflects maladaptive neuroplasticity of malleable neural networks, and people will revert back to learned and habitual physical inactivity patterns, particularly in the setting of stress or depression. We hypothesize that behavioral interventions that successfully promote physical activity may also enhance adaptive neuroplasticity and play a key role in the maintenance of physical activity through the development of new neuronal pathways that enhance functional ability in older adults. Conversely, without such adaptive neuroplastic changes, ingrained maladaptive neuroplasticity will prevail and long-term maintenance of physical activity will fail. In this paper we will: (1) describe the enormous potential for neuroplasticity in older adults; (2) review stress and depression as examples of maladaptive neuroplasticity; (3) describe an example of adaptive neuroplasticity achieved with a behavioral intervention that induced positive affect in people with CHD; and (4) discuss implications for future work in bench to bedside translational research.

## 1. Introduction

Physical activity is a seemingly simple, clinically potent and cost-effective approach to reduce morbidity and mortality, particularly in high-risk chronic disease populations, such as those with coronary heart disease (CHD). For example, people with CHD who are physically active have a 26–47% reduction in 2–5-year all-cause mortality and a 28–30% reduction in cardiac mortality [1–5]. Nonetheless, long-term maintenance of physical activity remains a frustratingly elusive goal for patients and practitioners alike. In this paper, we hypothesize that recidivism after the initiation of physical activity may represent maladaptive neuroplastic changes in malleable neural processes in older adults with CHD who do not possess the neuronal networks necessary to support maintenance of physical activity. It has previously been established that physical activity promotes adaptive neuroplastic changes in older human beings and thus, based on animal studies, this likely represents neurogenesis, enhanced synaptic plasticity and dendritic branching. We further hypothesize that behavioral interventions that successfully promote physical activity may also enhance adaptive neuroplasticity and play a key role in the maintenance of physical activity through the development of new neuronal pathways that enhance functional ability in older adults. Conversely, without such adaptive neuroplastic changes, ingrained maladaptive neuroplasticity will prevail and long-term maintenance of physical activity will fail.

State-of-the-art behavioral science research that is designed to motivate the initiation and maintenance of physical activity usually incorporates three core elements: (1) education; (2) behavioral modification (e.g., exercise instruction; and (3) cognitive-behavioral approaches (e.g., self-monitoring, behavioral goal setting) [6]. In combination, these approaches can effectively promote physical activity, and thus enhance adaptive neuroplasticity, in healthy community-dwelling older adults [7, 8]. However, previous efforts to motivate long-term maintenance of physical activity in older adults with chronic disease have been disappointing, particularly among people with CHD [6, 9, 10]. Behavioral science has not normally employed the concepts and dynamics of adaptive neuroplasticity when designing studies to promote behavioral change. Given the enormous potential for physical activity to yield improved health outcomes in people with CHD, it is imperative that investigators apply modern constructs of neurobiology to the development of more effective and enduring behavioral science treatment approaches. 

Translation of successes from basic behavioral science to high-risk, clinically ill patient groups has not traditionally been employed and holds great potential to motivate initiation and maintenance of behavior change and, in turn, improve long-term health outcomes. For example, induction of positive affect has demonstrated the ability to motivate both increased physical activity and improved medication adherence in two studies of chronic disease [11, 12]. Given their potential potency, the application of such approaches to motivate physical activity may also stimulate adaptive neuroplastic changes that can support long-term physical activity maintenance. One barrier to translational behavioral science has been the absence of a standardized approach to translate basic behavioral and social science research to the study and treatment of patients with chronic disease. To this end, we have recently developed EVOLVE, which is a mixed methods and staged approach for this purpose [13]. While generalizing from animal models to humans is always complicated, this new frontier of behavioral medicine holds great promise because it begins to adopt the neurobiologic concepts of adaptive neuroplasticity in its application to behavioral change in chronic disease. 

This paper will focus on the maladaptive neural pathways that lead to increased risk of adverse outcomes among people with CHD. We will (1) describe the great potential of neuroplasticity in older adults; (2) review stress and depression as examples of maladaptive neuroplasticity; (3) describe an example of adaptive neuroplasticity achieved with a behavioral intervention that induced positive affect in people with CHD; and (4) discuss implications for future work in bench to bedside translational research.

## 2. Neuroplasticity and Aging

The effect of aging on the brain has been well documented. This includes impaired memory [14], declines in neurocognitive functioning, diminishing numbers of neurons and synapses [15], physical and biologic changes that accompany behavioral impairments, and functional and physical deterioration over time [16]. While many have postulated that these changes are inevitable and part of the normal process of aging, there is growing literature in neuroscience and aging that focuses on the potential for both animals and humans to achieve adaptive neuroplastic changes in an activity-dependent manner through both physical activity and environmental enrichment [17, 18]. 

### 2.1. Overview of Adult Neurogenesis

Adult neurogenesis, the process of new neuron production and functional integration in the adult brain, occurs across a range of species, in both vertebrate and invertebrate animals [19, 20]. For example, virtually all mammals [21–24], including humans [22, 25] have been found to add functional neurons throughout life. In mammals, neurogenesis has been shown to occur in the amygdala [26, 27], the neocortex [27, 28], the olfactory bulb [25], the striatum [25] and the hypothalamus [26], among other areas. However, human neurogenesis occurs in two main areas of the brain: the subventricular zone (SV ZONE) lining the lateral ventricles, and the subgranular zone (SG ZONE) in the dentate gyrus of the hippocampus [29–31].

In the SV ZONE, it is hypothesized that astrocytes behave as slow-dividing neural cells and produce neuroblast precursors. The neuroblasts migrate in chains to the olfactory bulb along the rostral migratory stream at a rate of 30,000/day where they break off from their chains and mature into either granule cells or periglomerular cells [32]. In the SG ZONE of the dentate gyrus, astrocytes generate intermediate progenitors at a rate of 9,000/day [33]. The progenitors mature into granule neurons [34] of the dentate gyrus and develop axonal projections into the CA3 area and dendritic arbors into the molecular layer [35, 36]. A cacophony of intrinsic and extrinsic factors and neurotransmitters affect the development, integration and survival of new neurons. The rates of neurogenesis and apoptosis are linked in the hippocampus [37], and while the size of the brain does not increase, the number of neurons in the dentate gyrus may increase in adulthood [33]. 

### 2.2. Neuroplasticity and Aging

Traditionally viewed as static and generally degrading over time, the aging brain is now increasingly recognized as “plastic” and changeable. The adult hippocampus is able to add new neurons to the network as a result of physical or cognitive activity; exercise induces precursor cell proliferation and environmental enrichment enhances the survival of newborn neurons [38]. The adult brain is able to grow and transform in response to physical activity, with angiogenesis, neurogenesis, enhanced synaptic plasticity and dendritic branching [39]. According to the “neurogenic reserve” hypothesis [40], continued physical and cognitive activity maintains the potential for adult neurogenesis, thereby creating a pool of potentially recruitable neurons into a neurogenic reserve. Key predictors of neurogenesis are previous experience, activity and novelty of experience, all of which are believed to increase the rate of neurogenesis based on previous effort [38, 41]. 

### 2.3. How Do Stress and Depression Affect the Aging Brain?

Chronic stress leads to decreased cell proliferation, primarily in the SG Zone [42]. This is believed to be due to elevated glucocorticoid levels that result when the hypothalamic-pituitary-adrenal (HPA) axis is stimulated during stress. Stress also leads to dendritic atrophy in the hippocampus and medial prefrontal cortex [43, 44]. People with major depression have hippocampal volumes that are, on average, 19% smaller than those of matched controls [45]. Hippocampal atrophy is greater with longer duration of depression, independent of age [46]. It is not known if hippocampal loss precedes depression, and thus, is a risk factor for the development of depression [47]. An alternative hypothesis is that the hippocampal volume loss is caused by excess secretion of glucocorticoids found in people with depression, which in turn leads to neuronal death, regression of dendritic branching and impaired neurogenesis—all leading to a loss of brain volume in the hippocampus [47]. Glucocorticoids are directly toxic to hippocampal neurons, as well as indirectly toxic by impairing synaptic glutamate removal and calcium mobilization [47]. 

### 2.4. How Does Exercise Affect the Aging Brain?

Exercise has clearly been demonstrated to enhance neurogenesis throughout life [17]. Exercise upregulates brain-derived neurotrophic factor (BDNF), a mediator of neurogenesis, and BDNF mRNA in the dentate gyrus, the lumbar spinal cord, cerebellum and cerebral cortex [48, 49]. Further, there is now unequivocal evidence in humans that physical activity increases hippocampal volume [50, 51]. For example, Erickson randomized 120 older adults to 40 minutes of walking 3 days/week versus a stretching control for 1 year [50]. Participants began by walking 10 minutes and increased walking by 5-minute increments each week until 40 minutes was achieved. Exercise participants had several important results: (1) anterior hippocampal volume increased by 2% over 1 year; (2) improvements in spatial memory were related to increases in hippocampal volume; and (3) greater serum levels of BDNF were related to increased hippocampal volume. In contrast, the control group demonstrated a 1.4% decline in anterior hippocampal volume over the year. Of note, in the exercise group, the anterior hippocampal volume increased a mean of 0.03 cm^3^ in the initial 6 months and then doubled (0.06 cm^3^) in the latter 6 months of training. This effect may be due to a fitness curve, however, these data raise questions concerning potential additional enhancements to hippocampal volume with training modifications, such as increasing the duration, intensity and frequency of physical activity to conform with AHA/ACSM/ACC national guidelines [52, 53]. Future work in this area holds great promise for older adults. 

A study that randomized people with an indication for coronary angioplasty to exercise alone versus coronary angioplasty found that not only did the people who exercised have significantly fewer major adverse clinical events over two years (22% versus 38%, *P* = 0.039), but they also had significant reductions in C-reactive protein and interleukin-6 (IL-6) levels [54]. These findings suggest that physical activity plays an important role in reducing inflammation, which may be one pathway by which physical activity exerts its protective effects. In this manner, physical activity may act on inflammatory mediators of stress and depression and promote adaptive neuroplastic changes to the brain. 

Physical activity also enhances cognitive function in older adults. Physically active older adults demonstrate processing of event-related brain potentials similar to that of younger people doing the same task, which suggests that physical activity may speed cognitive performance and change the provision of attentional resources in older adults who exercise [55, 56]. Reviews have also found that physical activity improves executive function [57, 58], attention [59], cognitive speed [59] and memory [60].

## 3. Maladaptive Neuroplasticity

### 3.1. Stress and Maladaptive Neuroplasticity

Environmental stress leads to HPA axis stimulation, activating the hormonal stress response. In response to environmental stress, the hypothalamus releases corticotropin-releasing hormone, which causes the release of adrenocorticotropic hormone (ACTH) from the anterior pituitary gland. ACTH then provokes the secretion of glucocorticoids. Chronic stress leads to dendritic atrophy in the hippocampus in animal studies [43]. Dendritic spine atrophy in the medial prefrontal cortex has also been noted with stress [44]. 

#### 3.1.1. Allostasis and Allostatic Load

Allostasis is the body's ability to respond to environmental stressors by increasing blood pressure, heart rate, endocrine output and neural activity [61]. Allostatic load is caused by repeated overactivation and malfunction of numerous mediators including the sympathetic-adrenal-medullary (SAM) axis release of catecholamines (e.g., norepinephrine, epinephrine), HPA axis activation (e.g., glucocorticoids), impaired immune function and increased cardiovascular reactivity in response to environmental stress [61, 62]. While activation of the SAM and HPA axes are normal physiologic responses to environmental stress (i.e., the fight-or-flight response), chronic over-activation damages the physiologic systems that preserve homeostasis, leading to alterations in the physiologic system response ranges and chronic wear and tear [61]. Over time, these changes can lead to stress-related maladaptive disease [62, 63]. For example, stress-induced increases in glucocorticoid secretion along with physical inactivity can lead to insulin insensitivity, increased body fat and ultimately to atherosclerosis, as in CHD. Allostatic load is the long-term cumulative toll of environmental stress, resulting from inappropriately frequent or excessive responses across the body's physiologic systems [63]. With regard to the effect of allostatic load on the brain, HPA axis stimulation by environmental stress causes release of corticotropin-releasing hormone, corticotropin and cortisol. Adrenal steroids protect the brain from its neurochemical response to environmental stress [64]. However, animal studies of social stress and aging have found that adrenal steroids impair hippocampal formation, worsen existing ischemia in the brain and create a stress-related wear and tear effect on cerebral processes [65, 66]. It is believed that both glucocorticoid secretion and altered neural activity cause attrition of the neuronal structure, but the exact cellular mechanism is not known [61]. Ultimately, allostatic load in the brain leads to impaired physiologic control and cognitive dysfunction [61]. 

The conceptual model of allostatic load and disease is as follows: a person will process a stressful event as either threatening or not threatening, determined by factors including genetic predisposition, developmental stage, learning and social history. If the stressor is viewed as threatening, the person may engage in a patterned stress response that often includes self-damaging behaviors, such as smoking or drinking alcohol. Behaviors are accompanied by biological responses. Specifically, neural and neuroendocrine mediators act on the immune, cardiovascular and bodily tissues to increase susceptibility to disease. Over time, unfavorable social, behavioral, psychological and environmental factors lay a pathophysiologic foundation for disease in the body [61]. Maladaptive behavioral coping (e.g., smoking) may decrease stress in the short term, but at a cost of long-term behavioral and biologic damage. For example, a person who smokes will increase smoking behavior during times of stress. Over time, maladaptive coping behaviors lead to disease, morbidity and mortality. 

The effect of allostatic load on physical and cognitive functioning and cardiovascular risk was evaluated in the longitudinal MacAuthur Studies in over 700 adults aged 70–79 [63]. Allostatic load was measured with 10 measures of physiologic function, including systolic and diastolic blood pressure, hip-waist ratio, glycosylated hemoglobin, total cholesterol-HDL ratio, HDL cholesterol, urinary cortisol, urinary norepinephrine, urinary epinephrine and dehydroepiandrosterone sulfate (DHEA-S). Higher allostatic load predicted new incident cardiovascular events over 2.5 years of follow-up, with events increasing in a dose-dependent manner in the categories of low, medium and high allostatic load. In addition, higher allostatic load was associated with significantly worse physical and cognitive function as well as significantly increased risk for cognitive decline over 2.5 years [63]. 

**Figure 1:**
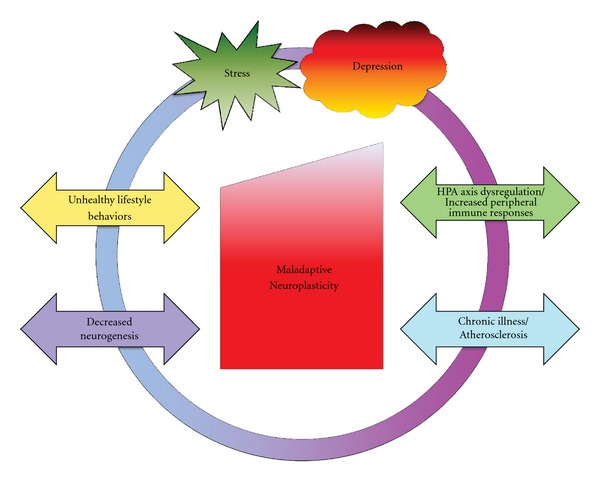
The Cycle of Maladaptive Neuroplasticity.

There are epidemiologic and clinical study data that also support this model. For example, in the INTERHEART study, people with greatest stress had significantly higher BMI and cholesterol, and were more likely to smoke (*P* < 0.0001 for all) [67]. In the Copenhagen City Heart Study, stress was inversely related to physical activity [68]. Thus, stressed people engage in unhealthy lifestyle behaviors. Several studies have found that men with high job demands had the greatest progression of carotid artery atherosclerosis over four years [69, 70]. In patients with unstable angina and myocardial infarction [71] and in healthy subjects [72], elevated levels of the acute-phase reactant C-reactive protein predicted adverse cardiovascular events and death in long-term follow-up. Coronary angioplasty patients demonstrate significantly elevated levels of C-reactive protein in response to mental challenges, indicating an exaggerated inflammatory response [73]. In another study of coronary angioplasty patients, those with elevated C-reactive protein levels had significantly increased rates of adverse cardiovascular events over 12 months (*P* < 0.001) [74]. In a study of 202 healthy men, those with elevated IL-6 levels had a 2.3-fold increased risk of myocardial infarction over 6 years [75]. In patients with unstable angina, those with elevated Il-6 levels had a 3.47-fold increased risk of death over 12 months [76]. In lab studies where stress was induced in healthy people, subjects' IL-6 levels were also increased after stress [77, 78]. Over time, these exaggerated responses are hypothesized to contribute to the development and progression of atherosclerotic disease through initiation of the inflammatory cascade. The Cycle of Maladaptive Neuroplasticity is depicted in Figure 1.

### 3.2. Depression and Maladaptive Neuroplasticity

People who are depressed have decreased hippocampal volumes [45]. Animal studies indicate that depression leads to decreased neurogenesis [79]. Post-mortem studies demonstrate that people suffering from long-term major depression have glial cell loss in the hippocampus [80], which could be a sign of dendritic retraction [81], as well as less glial density in the prefrontal cortex and cingulate cortex [82]. Chronically depressed people also demonstrate signs of glial cell loss in the amygdala [83]. Behaviorally, depression in older adults can be accompanied by executive dysfunction, including states such as behavioral and functional disability, psychomotor retardation, apathy and reduced insight [84]. 

#### 3.2.1. The Inflammation Hypothesis

The inflammation hypothesis in geriatric depression proposes that aging and increased comorbidity lead to: (1) heightened peripheral immune system responses; (2) a proinflammatory central nervous immune system; and (3) impaired communication between the peripheral immune system and central nervous immune system [85]. Abnormal immune responses mediate changes to the neural networks and predispose older adults to depression. According to the inflammation hypothesis, when vulnerable older adults with chronic disease are faced with an immune challenge, hypervigilant microglia release high amounts of IL-1*β*, IL-6 and tumor necrosis factor-*α* (TNF-*α*) [86]. Activation of microglia stresses the cells and leads to loss of neurons, impairs plasticity and reduces neurogenesis [86]. This results in changes consistent with the behavioral and cognitive symptoms seen in geriatric depression. These changes are mediated by metabolic brain changes [87], which are more likely to develop in people with brain abnormalities in vulnerable areas where emotional regulation occurs, specifically areas of cognitive control (dorsal anterior cingulate and lateral prefrontal cortex), emotional control (dorsal and rostral anterior cingulate and amygdala) and the hippocampus [85]. Aging or disease-related states promote metabolic changes in the brain which mediate the depressive syndrome and/or enhance brain abnormalities, and predispose older adults to depression [85]. 

Depression or depressive symptoms are a prevalent and debilitating form of maladaptive neuroplasticity affecting approximately 20–40% of people with CHD [88–90]. Depression and cardiovascular events have been widely studied in CHD; depressed people are more likely to develop CHD (meta-analytic effect size, 1.5–2.7) [91–94] and people with established CHD who are depressed have increased risk of morbidity and mortality (meta-analytic effect size, 1.6–2.2) [93, 95, 96]. 

People with symptoms of depression engage less in preventive health behaviors, which places them at higher cardiovascular risk. For example, in a study of 1,017 veterans with CHD, the association between depression and adverse events was largely explained by behavior, and in particular, physical inactivity [97]. Depression in older adults may be accompanied by executive dysfunction. Executive dysfunction appears to contribute to people's inability to manage risk factors, perhaps because they cannot problem-solve and experience attentional impairment, psychomotor slowness or impaired self-control. The mechanism underlying depression and adverse clinical outcomes is maladaptive neuroplasticity. Proinflammatory cytokines and immune mediators that give rise to depression also stimulate HPA-axis hyperactivity [98–101]. People with major depression have higher levels of IL-6, IL-1, C-reactive protein and TNF-*α* [102–104].

## 4. Adaptive Neuroplasticity

**Figure 2:**
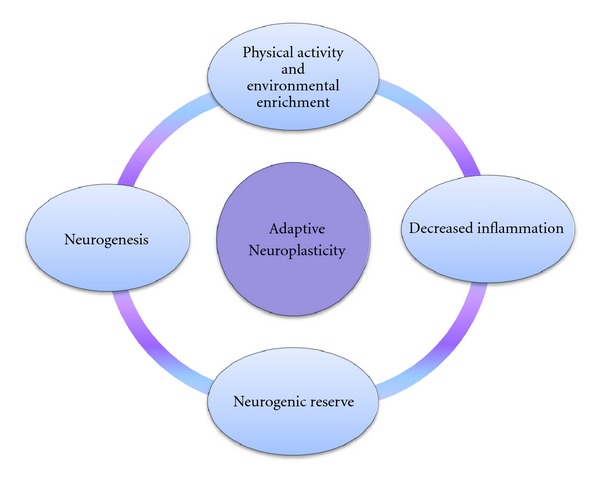
The Cycle of Adaptive Neuroplasticity.

A compelling literature exists supporting physical activity and its effects on adult functional neuroanatomy. Exercise has been shown to lead to robust increases in neurogenesis and enhanced learning in aged animals [17, 105] and the mechanism by which this occurs is through both increased precursor cell proliferation and survival [106, 107]. In fact, even low levels of physical activity appear to induce cell proliferation [108]. It is hypothesized that new neurons are integrated into the neuronal network in a manner that can lead to lasting changes to the network [38]. Activity-dependent adaptations are made to the mossy fiber connection between the dentate gyrus and the CA3 area based on prior levels of experience [109]. Exercise also upregulates BDNF protein and BDNF mRNA [48, 49]. Further, there is strong evidence in humans that physical activity increases hippocampal volume [50, 51]. Therefore, it is clear that exercise reorganizes the brain. The Cycle of Adaptive Neuroplasticity is depicted in Figure 2.

### 4.1. Case Study: Translational Behavioral Research to Motivate Physical Activity

Given the great challenge in promoting long-lasting physical activity in people with CHD, novel approaches must be evaluated. To this end, recent work has translated a basic behavioral science intervention, induction of positive affect, to CHD patients [13, 110]. Positive affect is a feeling of satisfying engagement, happiness and contentment [111]. Induction of positive affect has shown potent and robust effects in many studies conducted in non-clinical populations [112]. These studies have shown that seemingly modest positive affect inductions produce powerful effects on people's behavior and thought processes. For example, positive affect promotes creativity and flexibility in problem-solving [113, 114] and leads people to feel more intrinsically motivated [115]. In addition, people in a state of positive affect are more accepting of trying new things and demonstrate greater interest in leisure activities [116, 117]. Positive affect also helps people engage in healthier behaviors or take precautions [118]. Given these results, induction of positive affect had high potential to promote behavior change in people with chronic illness. 

**Figure 3:**
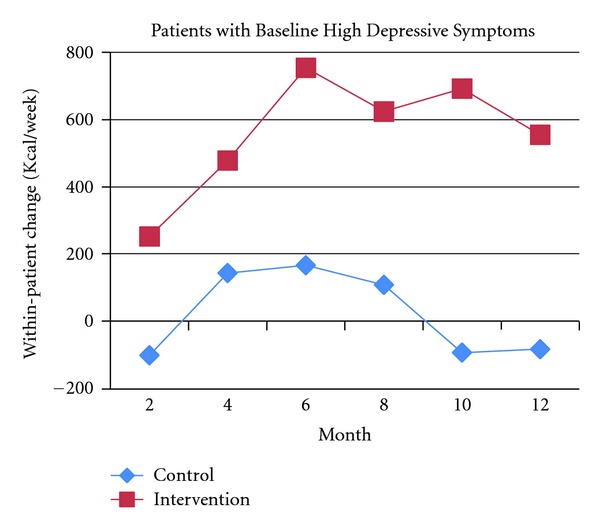
Physical activity expenditure over 12 months in the positive affect intervention versus control group among patients with high depressive symptoms at baseline *P = 0.038 at 12 months.

**Figure 4:**
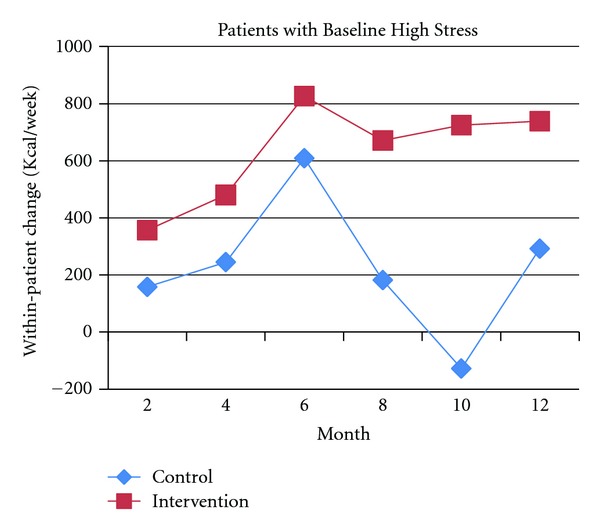
Physical activity expenditure over 12 months in the positive affect intervention versus control group among patients with high stress at baseline.

A recent RCT tested positive affect induction versus an educational control in 242 post-coronary angioplasty patients to motivate increased physical activity over 12 months [11]. This study found that the positive affect intervention group had nearly double the improvement in within-patient kilocalorie (kcal)/week expenditure at 12 months when compared to controls (602 versus 328 kcal/week, *P* = 0.027) [11]. This expenditure is equivalent to the positive affect participants walking 7.5 miles/week versus the control participants walking 4.1 miles/week. In addition, the positive affect intervention was effective in motivating physical activity among participants with high depressive symptoms or stress at baseline (Figures 3 and 4). Participants with high depressive symptoms at baseline (score of ≥10 on the 10-item CES-D [119, 120]) who were in the positive affect intervention group were able to achieve significant increases in kcal/week expenditure at 12 months compared to controls (561 versus -61 kcal/week, *P* = 0.038) (Figure 3). Participants with high stress at baseline (score of ≥20 on the 20-item Perceived Stress Scale [121]) who were in the positive affect intervention condition also tended to achieve higher (but not statistically significant) within-patient kcal/week increases in physical activity over 12 months compared to the control group (Figure 4). 

The hypothesis being advanced in this paper is that the link between positive affect induction and greater adherence to exercise is mediated by changes in the nervous system, exemplifying adaptive plasticity. These findings demonstrate that induction of positive affect motivated CHD patients to initiate and maintain physical activity, even in the face of stress and depression, thereby assisting them to escape from the Cycle of Maladaptive Neuroplasticity (Figure 1) and begin to employ components of the Cycle of Adaptive Neuroplasticity (Figure 2). 

## 5. Discussion

Adult-generated neurons in the dentate gyrus of the hippocampus are stimulated by physical activity. This holds great potential for people with CHD, in whom physical activity decreases the risk of future morbidity and mortality. Remarkably, while there is a large literature on rehabilitation and learning, along with subsequent brain-plastic adaptations that support behavior in both animal models and humans, this same neuroscience has not been applied to behavioral science interventions to motivate people with high-risk chronic disease to initiate and maintain new health behaviors (i.e., physical activity). Given the complexity of chronic illness, it is imperative that basic biological, psychological and clinical researchers work together to solve complex issues facing modern medicine. Physical activity is a proven and effective approach to decrease cardiovascular risk and extend life. In addition, physical activity enhances adult neurogenesis and learning. Nonetheless, most older adults with chronic disease simply do not engage in regular physical activity. There is great unrealized potential to apply the constructs of adaptive neuroplasticity to promote long-term behavior change in high risk patient groups. 

### 5.1. Does Induction of Positive Affect Trigger Adaptive Neuroplasticity?

We have shown that induction of positive affect can significantly increase physical activity expenditure over 12 months in post-coronary angioplasty patients compared to a control group [11]. In this paper, we have conceptualized our positive affect intervention as the retraining of neural networks, which counter the downward spiral of degraded brain function in the aging brain of CHD patients. Importantly, this intervention also enabled participants with high levels of depressive symptoms or stress to achieve important increases in physical activity over 12 months. The mechanisms by which positive affect influences health have been hypothesized to occur though neuroendocrine, inflammatory and immune mediators [122]. Studies have found that people in positive affect states have lower levels of salivary cortisol [122, 123], decreased inflammatory mediators (C-reactive protein [124] and IL-6 [123, 124]) and enhanced heart rate variability [125]. 

It has also been hypothesized that positive affect stimulates the release of dopamine [126]. This assertion fits well with what is known about adult neuroplasticity. The SV ZONE is innervated by dopaminergic fibers that originate in the substantia nigra, and the SG ZONE is innervated by dopaminergic fibers that originate from the ventral tegmental area [127]. Proliferation of neural progenitors in both the SV ZONE and the SG ZONE have been shown to be controlled by dopaminergic signaling [128]. Ashby and Isen postulated that induction of positive affect leads to dopaminergic stimulation of the anterior cingulate region via mesocorticolimbic projections and stimulation of the striatum via subtantia nigra projections [126]. The mesocorticolimbic system in the ventral tegmental area is associated with reward, motivation and pleasant feelings, while the nigrostriatal system in the substantia nigra is known to be associated with motor activity and specific cognitive tasks [126]. Neuroplasticity in these areas may explain why people are more motivated to engage in physical activity as a result of positive affect induction. 

### 5.2. What Other Approaches Might Promote Adaptive Neuroplasticity?

It has been suggested that interventions that focus on prosocial interactions as well as indirect approaches to enhance well-being may also induce neuroplastic changes in the brain [129]. Interventions such as cognitive therapy, social service programs for the elderly and meditation may all promote neuroplasticity in order to achieve their behavioral effects [129]. Another interesting intervention that is conceptualized as promoting adaptive neuroplasticity is Mellin's Emotional Brain Training [130–133], which is an educational counseling approach aimed at equipping individuals with self-regulatory tools to promote stress-related coping and relearn strategies to achieve low stress states, healthy coping and lifestyle skills. Thus, there appear to be a variety of behavioral approaches that may promote plasticity. 

## 6. Conclusions

Physical activity promotes neuroplasticity in both animals and older adults. Further, physical activity decreases morbidity and mortality in people with CHD. The lack of evidence-based approaches to effectively motivate long-term initiation and maintenance of physical activity is a pressing public health issue for people with CHD. The issue of how to motivate maintenance of adaptive neuroplasticity and whether neuroplasticity can support maintenance of physical activity through the development of new neural networks has not been studied. To this end, approaches to assess neural networks are being developed, which could assist in identifying pathways that support maintenance of physical activity in high risk patients. While methods to measure grey matter density changes as a result of physical activity are available and used in humans [50], critical structural changes, neural networks and connectivity in white matter are areas that are just beginning to be explored [134]. 

In conclusion, the optimal timeframe necessary to achieve maximal neuroplastic brain changes remains unknown, as do the optimal activity maintenance patterns required to sustain neuroplastic changes in humans. Whether neuroplasticity supports maintenance of physical activity through the establishment of new neural networks, as hypothesized in this paper, is also unexplored. Finally, it has been suggested that critical or sensitive timeframes may exist for plasticity in response to interventions, and this should also be evaluated [129].

## 7. Directions for Future Practice

1. The asymptote of adaptive neuroplastic brain changes associated with new behavioral change (i.e., physical activity) remains unknown and likely varies widely according to individual genetic, environmental, behavioral and physiologic factors. Studies exploring this issue would greatly assist in defining the optimal “dose” and most favorable types of behavioral interventions for older adults with chronic illness. This is particularly true for studies of physical activity interventions in high-risk older adults, which may require longer reinforcement before habits are maintained and risk reduction benefits are achieved.
2. Interventions that can be self-reinforced and include individually derived, personally-relevant information are important in motivating behavioral maintenance because they are tailored and can be implemented and drawn upon by people outside of the study setting [13, 110].
3. Positive affect induction in clinical populations is an important approach to motivate the initiation and maintenance of behavior change and, we hypothesize, promotes adaptive neuroplasticity [11, 110]. Other approaches exist that may also trigger adaptive neuroplasticity and these should also be evaluated.
4. Behavioral interventions should focus on the underlying etiology of maladaptive neuroplasticity. Beginning in the design phase, the ultimate goal of a new intervention is that it will be efficacious, effective and broadly appealing. Interventions that have limited uptake (e.g., in small, highly motivated subgroups) should be carefully evaluated from a cost-benefit perspective prior to proceeding.
5. Behavioral science interventions should include multidisciplinary teams of clinical epidemiologists, neuroscientists, psychologists and behavioral scientists in order to assure that concepts of neuroplasticity are incorporated into the study design and rigorously assessed longitudinally.
6. Behavioral interventions that combine physical activity and cognitive stimulation have not been widely evaluated, but may offer synergistic benefits, thereby building on the “neurogenic reserve hypothesis” [38, 40].
